# Utility of cerebrovascular imaging biomarkers to detect cerebral amyloidosis

**DOI:** 10.1002/alz.14207

**Published:** 2024-09-01

**Authors:** Matthew D. Howe, Megan R. Caruso, Masood Manoochehri, Zachary J. Kunicki, Sheina Emrani, James L. Rudolph, Edward D. Huey, Stephen P. Salloway, Hwamee Oh, for the Alzheimer's Disease Neuroimaging Initiative

**Affiliations:** ^1^ Butler Hospital Memory & Aging Program Providence Rhode Island USA; ^2^ Department of Psychiatry and Human Behavior Brown University Providence Rhode Island USA; ^3^ University of Pennsylvania Perelman School of Medicine Philadelphia Pennsylvania USA; ^4^ Center of Innovation in Long‐Term Services and Supports, Providence VA Medical Center Providence Rhode Island USA; ^5^ Department of Medicine The Warren Alpert Medical School of Brown University Providence Rhode Island USA

**Keywords:** ADNI, Alzheimer's disease, amyloid beta, cerebrovascular disease, magnetic resonance imaging, positron emission tomography, small vessel disease

## Abstract

**INTRODUCTION:**

The relationship between cerebrovascular disease (CVD) and amyloid beta (Aβ) in Alzheimer's disease (AD) is understudied. We hypothesized that magnetic resonance imaging (MRI)–based CVD biomarkers—including cerebral microbleeds (CMBs), lacunar infarction, and white matter hyperintensities (WMHs)—would correlate with Aβ positivity on positron emission tomography (Aβ‐PET).

**METHODS:**

We cross‐sectionally analyzed data from the Alzheimer's Disease Neuroimaging Initiative (ADNI, *N* = 1352). Logistic regression was used to calculate odds ratios (ORs), with Aβ‐PET positivity as the standard‐of‐truth.

**RESULTS:**

Following adjustment, WMHs (OR = 1.25) and superficial CMBs (OR = 1.45) remained positively associated with Aβ‐PET positivity (*p* < 0.001). Deep CMBs and lacunes exhibited a varied relationship with Aβ‐PET in cognitive subgroups. The combined diagnostic model, which included CVD biomarkers and other accessible measures, significantly predicted Aβ‐PET (pseudo‐R^2 ^= 0.41).

**DISCUSSION:**

The study highlights the translational value of CVD biomarkers in diagnosing AD, and underscores the need for more research on their inclusion in diagnostic criteria. **ClinicalTrials.gov**: ADNI‐2 (NCT01231971), ADNI‐3 (NCT02854033).

**Highlights:**

- Cerebrovascular biomarkers linked to amyloid beta (Aβ) in Alzheimer's disease (AD).
- White matter hyperintensities and cerebral microbleeds reliably predict Aβ‐PET positivity.
- Relationships with Aβ‐PET vary by cognitive stage.
- Novel accessible model predicts Aβ‐PET status.
- Study supports multimodal diagnostic approaches.

## 1 BACKGROUND

Alzheimer's disease (AD), the leading cause of dementia worldwide, is biologically defined by the presence of amyloid beta (Aβ) plaques and tau neurofibrillary tangles.^1^, ^2^ Although understudied, neuropathological and neuroimaging studies indicate that cerebrovascular disease (CVD) is a common finding in patients with AD, with AD and CVD co‐pathology accounting for ≈20%–30% of cases in some cohorts.^3^, ^4^, ^5^ The rising incidence of AD, attributed partly to increased life expectancy, is also driven by modifiable CVD risk factors, including hypertension, diabetes mellitus, and hyperlipidemia.^6^, ^7^, ^8^ These modifiable CVD risk factors have been shown to interact with age, sex, and apolipoprotein E(*APOE*) ε4 genotype, potentially influencing susceptibility to AD via actions on glial cells, changes in blood–brain barrier structure/function, and decreased perivascular clearance of Aβ peptides in animal models.^7^, ^9^, ^10^, ^11^ Furthermore, CVD may promote perivascular Aβ deposition that exerts downstream effects on the neurovascular unit (NVU), including basement membrane fibrosis, perivascular inflammation, and Aβ‐mediated vasoconstriction—with potential additive and synergistic effects on cognitive decline in AD.^11^, ^12^, ^13^, ^14^ Therefore, the relationship between CVD and AD may be bidirectional along the progression of pathology and clinical severity.

Much interest has been paid to using structural neuroimaging to identify small vessel disease and vascular dementia in vivo, with radiopathologic studies indicating that structural magnetic resonance imaging (MRI) features of CVD, including cerebral microbleeds (CMBs) and white matter hyperintensities (WMHs), are predictive of perivascular Aβ plaques and cerebral amyloid angiopathy (CAA) on autopsy.^2^, ^7^, ^15^, ^16^, ^17^ The use of CVD measures to aid in detecting total Aβ burden in AD (i.e., parenchymal and vascular plaques) is understudied,^13^ but could be applied clinically to aid in selecting patients for testing with positron emission tomography (Aβ‐PET) or interpreting the results of novel blood biomarkers. However, prior to clinical use, there is a need to characterize the potential utility of CVD biomarkers in identifying Aβ pathology and estimating AD risk in various stages of cognitive decline, which may vary given the potential additive burden of CVD and AD pathology on cognition.^5^, ^7^

Seeking to identify a cerebrovascular “signature” of Aβ pathology in AD, we investigated the degree to which CVD neuroimaging biomarkers—including CMBs, lacunar infarction, and WMHs—correlate with Aβ‐PET positivity, a core biomarker of AD under the Amyloid/Tau/Neurodegeneration framework.^2^ We selected these CVD biomarkers due to: (1) high‐quality neuropathological evidence linking them to relevant age‐related cerebral small vessel diseases and/or neuropathology of AD (i.e., Thal, Braak, or Consortium to Establish a Registry for Alzheimer's Disease [CERAD] staging)^16^; (2) the utility of incorporating these biomarkers into existing algorithms for small vessel disease detection, such as the Boston Neuroimaging Criteria 2.0 or the Cerebral Small Vessel Disease Score^4^, ^15^, ^17^; as well as (3) relative ease of assessment with clinical MRI protocols that are already used in health care settings.^18^ Based on the existing literature, we hypothesized that these CVD biomarkers would exhibit independent, positive correlations with Aβ‐PET positivity, and further sought to explore how their inclusion, alongside other accessible clinical, genetic, and neuroimaging measures, might improve prediction of Aβ‐PET positivity in AD cases and controls.^19^, ^20^, ^21^, ^22^, ^23^, ^24^

To test these hypotheses, we used participant data from the Alzheimer's Disease Neuroimaging Initiative (ADNI), a well‐described observational cohort study of older adults with AD.^25^, ^26^ Taking advantage of this cohort enriched for AD, we primarily sought to characterize the associations between commonly identified MRI‐visible CVD measures and Aβ‐PET positivity. We further explored the diagnostic performance of an accessible algorithm, which includes demographic (age, sex), genetic (apolipoprotein E [*APOE*] ε4 status), cognitive (Montreal Cognitive Assessment [MoCA]), and MRI‐visible CVD measures.^25^, ^26^ To inform future studies in specific clinical populations, we additionally stratified participants into cognitively unimpaired (CU), mild cognitive impairment (MCI), and dementia cohorts for further analyses.

## 2 METHODS

### 2.1 Participants

The ADNI was launched in 2003 as a public‐private partnership, led by Principal Investigator Michael W. Weiner. This study utilizes data obtained from ADNI‐2 (NCT01231971) and ADNI‐3 (NCT02854033), two recent phases of this landmark study that has collected data at over 50 sites in North America and has been used to develop clinical, imaging, genetic, and biochemical biomarkers for the early detection and tracking of AD over the past 20 years (adni.loni.usc.edu).^25^, ^26^ The primary goal of ADNI has been to test whether serial MRI, PET, other biological markers, as well as clinical and neuropsychological assessments can be combined to measure the progression of MCI and early AD.

Participants in ADNI are 55–90 years of age, and either English or Spanish speaking. Exclusions include inability to tolerate a blood draw, contraindication to neuroimaging, evidence of infarction or other focal brain lesion (those with multiple lacunes or lacunes in a critical memory structure are excluded), any significant neurologic disease, major recent psychiatric disorders, or significant systemic illness or medical conditions. Baseline diagnoses were determined using a combination of cutoff scores from several instruments including the Mini‐Mental Status Examination (MMSE), Cognitive Change Index (CCI), Weschler Logical Memory Test, and Clinical Dementia Rating (CDR) Staging Instrument. Based on these assessments, participants were assigned a diagnostic category according to expert consensus of cognitively normal (CN), subjective memory complaint (SMC), mild cognitive impairment (MCI), and AD dementia. Written informed consent was obtained from all participants or authorized representatives prior to the start of study procedures, with local institutional review boards and research ethics boards providing oversight for the study. The authors certify that the study was performed in accordance with the ethical standards as laid down in the 1964 Declaration of Helsinki and its later amendments.

### 2.2 ADNI data set

For the current analysis, we downloaded demographic, neuroimaging, and genetic data from the Laboratory of Neuro‐Imaging (LONI) site where ADNI data is housed, including Aβ‐PET, structural MRI, and *APOE* genotype determined by the Illumina Human BeadChip panel.^27^ Due to differences in imaging protocols and PET tracers in ADNI‐1, we limited our analysis to participants who screened for ADNI‐2 and ADNI‐3 (*N* = 1430). After a review of screening and baseline visit data, 78 individuals with unknown Aβ‐PET status (*N* = 76) or absent clinical diagnosis (*N* = 2) were excluded to yield our final sample (*N* = 1352).

**RESEARCH IN CONTEXT**

- **Systematic review**: Our comprehensive review of the Alzheimer's disease (AD) radiopathologic literature underscored that although cerebrovascular biomarkers are well documented in small vessel disease, their individual and potentially additive effects in detecting amyloidosis are understudied. This highlighted the need for a focused study on how these biomarkers operate across different stages of AD, particularly as part of a multimodal diagnostic strategy.
- **Interpretation**: Our research newly demonstrates the unique and independent effects of cerebrovascular biomarkers in detecting amyloidosis in various cognitive stages of AD. This contribution improves the current understanding of specific cerebrovascular biomarkers across the AD spectrum, revealing nuanced relationships between these biomarkers and the disease.
- **Future directions**: Future studies should seek to examine how location‐specific cerebrovascular factors affect cognitive and functional decline over time, explore integration with blood biomarkers to enhance diagnostic accuracy, and establish their validity across diverse populations in cohorts with vascular and other non‐AD dementias.

Data frames were downloaded from LONI using the *adnimerge* R package *(version 0.1.1)*. We obtained up‐to‐date demographic, cognitive, and *APOE* genotypes from the Alzheimer's Disease Cooperate Study *(ADNIMERGE)*. In addition, we obtained imaging data including whole and regional brain volumes from University of California at San Francisco *(ADNIMERGE)*, WMH and infarct data from the Center for Neuroscience at University of California at Davis *(UCD_WMH_05_02_22, MRI_INFARCTS_01_29_21)*, region‐specific CMB counts from the Mayo Clinic Aging and Dementia Imaging Research Laboratory *(MAYOADIRL_MRI_MCH_09_07_22)*, and florbetapir and florbetaben standardized uptake value ratio (SUVR) summary data from the University of California at Berkley *(UCBERKELEYAV45_04_26_22, UCBERKELEYFBB_04_26_22)*. For a detailed summary of the standard imaging acquisition and analysis pipelines used in ADNI, see Supplementary Methods. We last accessed the ADNI database on December 27, 2023.

### 2.3 Determination of Aβ positivity

Participants were classified as Aβ positive or Aβ negative based on Aβ‐PET SUVRs according to the standard analysis pipeline at the University of California at Berkley (see Supplementary Methods). Aβ‐PET positivity was defined as SUVR >1.11 and SUVR >1.08 for florbetapir and florbetaben, respectively.^28^, ^29^ For quantitative comparisons, SUVR was transformed to a standardized Centiloid scale as described previously by Royse et al.^30^

### 2.4 Quantification of medial temporal lobe and white matter volumes

Using T1 and T2‐weighted MRI images, WMH and medial temporal lobe (MTL) volumes were quantified by the Center for Neuroscience at University of California at Davis and the University of California at San Francisco, respectively, according to the standard FreeSurfer analysis pipeline (See Supplementary Methods). To control for inter‐individual and disease stage–related variability in global atrophy, mean MTL and total WMH volumes (WMHv) were normalized to whole brain volumes, and then log transformed.

### 2.5 Quantification and localization of CMBs

“Definite” or “probable” CMBs were identified and reported by the Mayo Clinic Aging and Dementia Imaging Research Laboratory by review of T2* gradient recall echo images according to the standard analysis pipeline (see Supplementary Methods). We divided CMBs into lobar (cortical and cerebellar) or deep (subcortical, periventricular white matter, and brainstem regions) as described previously by Charidimou et al.^15^ We then classified participants by the presence or absence of superficial and deep CMBs, respectively.

### 2.6 Quantification of lacunar infarcts

Lacunar infarcts were identified by a physician specifically trained in interpretation of MRI, with infarct size, location, and other imaging characteristics recorded according to the standard analysis pipeline at the Center for Neuroscience at University of California Davis (see Supplementary Methods). For this analysis, we considered only lesions equal to or greater than 3 mm in size as cerebral infarcts. We then classified participants based on the presence or absence of infarcts.^31^

### 2.7 Covariates

We adjusted all models for demographics (age, sex), cognition (MoCA score), and *APOE* genotype (number of ε4 alleles).

### 2.8 Statistics

Data cleaning, analysis, and plotting were conducted in R using the *mice, psfmi, pROC*, and *ggplot2* packages. Missing data comprised no more than 5% of the overall sample and were handled by multiple imputation by chained equations (Table S1). Descriptive statistics were calculated for each group and reported as mean (SD), median (IQR), and *N* (%) according to Rubin's rules.^32^ Adjusted odds ratios (ORs) were calculated by logistic regression with dichotomized Aβ‐PET status as the outcome and reported with a 95% confidence interval (CI), with statistical significance determined by the Wald test. For evaluation of regression model goodness‐of‐fit and parsimony, we used prediction model pooling, selection, and performance evaluation across multiply imputed data sets to calculate the Nagelkerke Pseudo‐R^2^ and Akaike information criterion (AIC). The Hosmer‐Lemeshow test was used to test goodness‐of‐fit compared to the null model, with *p* > 0.05 denoting acceptable model fit to the observed data.

Exploratory receiver‐operating characteristic (ROC) analysis was performed to calculate area under the curve (AUC) as well as diagnostic sensitivity, specificity, and accuracy at Youden's optimal cutoff. Nested models were created using forward variable selection: Step 1: Base (age, sex, *APOE* ε4 genotype), Step 2: Base + MTLv, and Step 3: Base +MTLv + CVD (see Table S2 for further details on model components). Nested models with forced forward selection were compared using the likelihood ratio test for multiply imputed data, reported as the d_3_ statistic with degrees of freedom (df) corresponding to difference in number of predictors between model steps. Statistical significance was defined by a threshold of *p* < 0.05.

## 3 RESULTS

### 3.1 Participant characteristics

To assess the relationships between CVD biomarkers and the outcome (Aβ‐PET positivity), we analyzed cross‐sectional screening visit data for ADNI‐2 and ADNI‐3 (*N* = 1352), and then subdivided participants based on cognitive stage at screening (Table 1). For the purposes of our analysis, participants categorized at their screening visit as cognitively normal or experiencing SMCs were classified as cognitively unimpaired (CU, *N* = 611), encompassing 198 (32.4%) Aβ‐PET positive and 413 (67.6%) Aβ‐PET negative participants. The MCI cohort (*N* = 531) contained 294 (55.4%) Aβ‐PET positive and 237 (44.6%) Aβ‐PET negative participants. Finally, the dementia cohort (DEM, *N *= 210) was predominantly Aβ‐PET positive (*N* = 181 [86.2%]), with a smaller proportion Aβ‐PET negative participants (*N* = 29 [13.8%]) (Table 1, Table S1).

**TABLE 1:** Baseline demographic and clinical characteristics.

	Cognitively unimpaired		Mild cognitive impairment		Dementia	
Characteristic^a^	Aβ‐ (*N* = 413)	Aβ+ (*N* = 198)	Aβ‐ (*N* = 237)	Aβ+ (*N* = 294)	Aβ‐ (*N* = 29)	Aβ+ (*N* = 181)
Age, years	70 (6)	73 (7)	70 (8)	73 (7)	76 (8)	74 (8)
Sex, male	184 (45%)	72 (36%)	134 (57%)	160 (54%)	24 (83%)	100 (55%)
Education, years	16.78 (2.34)	16.53 (2.48)	16.35 (2.46)	16.29 (2.61)	16.62 (2.24)	15.51 (2.61)
*APOE* ε4, alleles						
* 0*	*313 (77%)*	*97 (49%)*	*180 (78%)*	*91 (31%)*	*23 (79%)*	*47 (26%)*
* 1*	*87 (21%)*	*89 (45%)*	*46 (20%)*	*151 (52%)*	*6 (21%)*	*88 (49%)*
* 2*	*6 (1.5%)*	*11 (5.6%)*	*5 (2.2%)*	*51 (17%)*	*0 (0%)*	*43 (24%)*
MoCA, score	26.05 (2.58)	25.65 (2.51)	23.7 (3.0)	22.5 (3.3)	18.2 (4.0)	16.9 (4.6)
MTLv, normalized (log)	−4.21 (0.10)	−4.22 (0.10)	−4.23 (0.10)	−4.28 (0.11)	−4.31 (0.13)	−4.38 (0.12)
WMHv, normalized (log)	−6.84 (1.33)	−6.24 (1.42)	−6.47 (1.40)	−6.03 (1.36)	−6.02 (1.01)	−5.69 (1.13)
CMBs, any location (1+)	84 (21%)	47 (24%)	58 (25%)	95 (32%)	6 (21%)	73 (41%)
CMBs, superficial (1+)	61 (15%)	42 (21%)	45 (19%)	82 (28%)	5 (18%)	66 (37%)
CMBs, deep (1+)	27 (6.6%)	10 (5.1%)	12 (5.1%)	27 (9.2%)	1 (3.6%)	18 (10%)
Infarct (1+)	24 (6.8%)	22 (12%)	17 (8.7%)	23 (9.1%)	1 (4.3%)	7 (4.5%)
Aβ‐PET burden, cl^b^	4 (9)	55 (31)	1 (10)	73 (33)	−3 (14)	88 (30)

*Note*: See Table S1 for missing values.

Abbreviations: APOE, Apolipoprotein E.; CMBs, Cerebral Microbleeds; MoCA, Montreal Cognitive Assessment; MTLv, Medial emporal Lobe Volume; WMHv, White Matter Hyperintensity Volume.

^a^ Mean (SD); *n* (%); Median (IQR).

^b^ CL, centiloids.

### 3.2 Development of the adjusted model for prediction of Aβ‐PET positivity

We next sought to gauge the relationship between specific MRI markers of CVD neuropathology and Aβ‐PET positivity in the overall cohort using a model that was adjusted by accessible measures of vascular risk (age, sex, *APOE* ε4), cognitive impairment (MoCA), and AD‐related atrophy (MTL volume [MTLv]) (Table 2). After adjustment for these covariates, we identified statistically significant effects of MRI‐based CVD biomarkers. We found that increased WMHv (OR = 1.25 [95% CI: 1.20–1.29], *p* < 0.001) and presence of one or more superficial CMBs (OR = 1.45 [95% CI: 1.31–1.61], *p* < 0.001) as being associated with Aβ‐PET positivity in the overall cohort (Table 2). We did not identify any statistically significant relationships between deep CMBs (OR = 0.88 [95% CI: 0.74–1.04], *p* = 0.12) or lacunar infarction (OR = 1.01 [95% CI: 0.89–1.15], *p* = 0.40) and Aβ‐PET positivity in the overall cohort (Table 2). The adjusted model exhibited a pseudo‐R^2^ = 0.41, consistent with a large effect size for goodness‐of‐fit.

**TABLE 2:** Prediction of Aβ‐PET positivity in the overall cohort.

Characteristic	OR^a^	95% CI^b^	*p*‐value
Age, years	1.05	1.04, 1.06	<0.001
Sex, male	0.66	0.60, 0.71	<0.001
*APOE* ε4, alleles	5.66	5.24, 6.11	<0.001
MoCA, score	0.89	0.88, 0.90	<0.001
MTLv, normalized (log)	0.12	0.08, 0.17	<0.001
WMHv, normalized (log)	1.25	1.20, 1.29	<0.001
CMBs, superficial (1+)	1.45	1.31, 1.61	<0.001
CMBs, deep (1+)	0.88	0.74, 1.04	0.12
Infarction (1+)	1.01	0.89, 1.15	0.90

Abbreviations: APOE, Apolipoprotein E; CMBs, Cerebral Microbleeds; MoCA, Montreal Cognitive Assessment; MTLv, Medial emporal Lobe Volume; PET, Positron Emission Tomography; WMHv, White Matter Hyperintensity Volume.

^a^ OR = Odds ratio.

^b^ CI, Confidence interval.

*N* = 1352.

Based on these findings, we next sought to use this model to understand how the relationships between CVD biomarkers and Aβ‐PET positivity may differ across different cognitive stages. Thus we repeated this regression analysis in the CU, MCI, and DEM cohorts described in the following sections, which are organized by CVD biomarker of interest.

### 3.3 Cerebral microbleeds

**FIGURE 1 alz14207-fig-0001:**
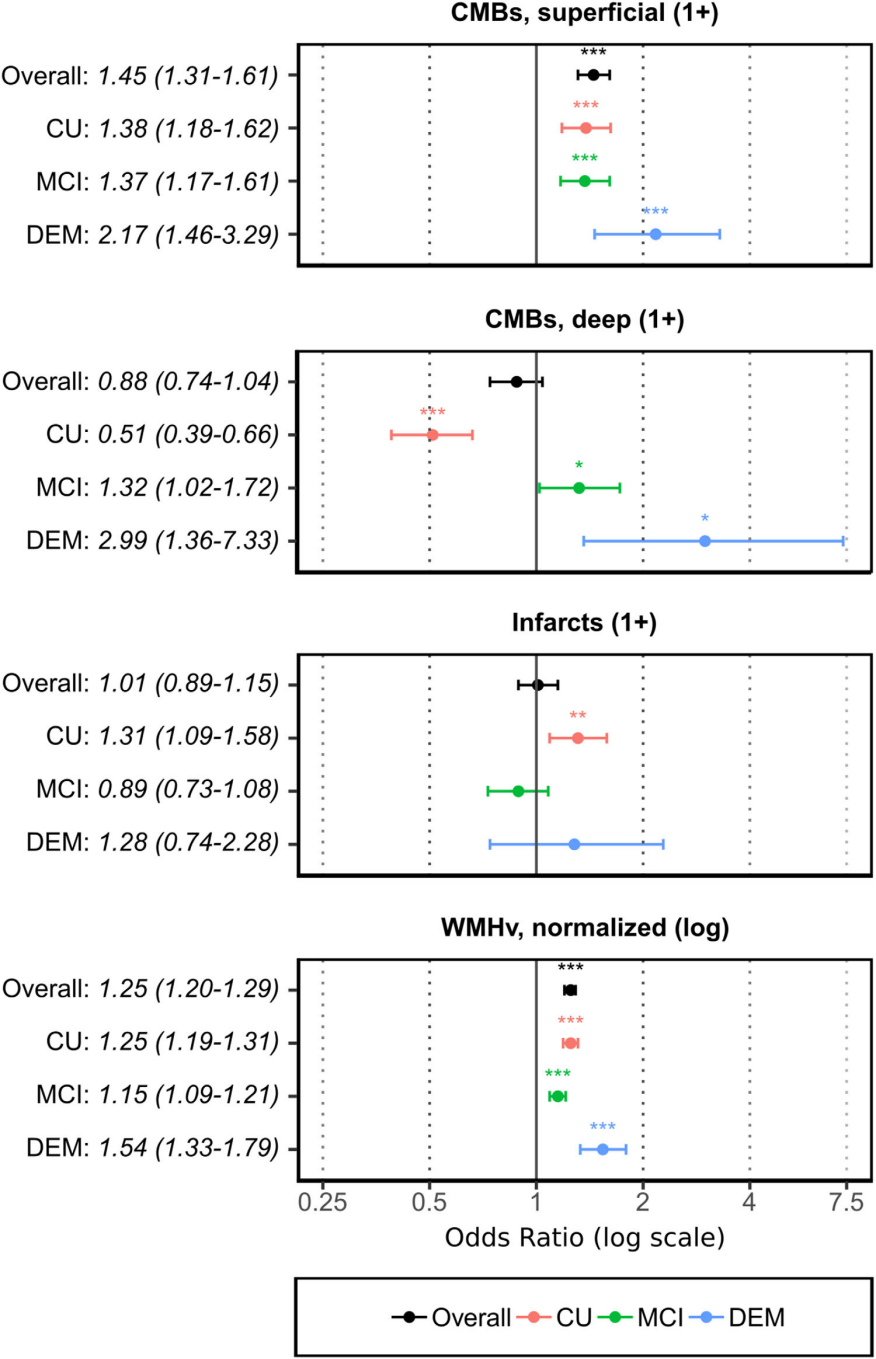
Differential prediction of Aβ‐PET positivity by CVD‐related MRI findings. Forest plots show the adjusted odds ratios (ORs) with 95% confidence intervals (CIs) for each cohort: Cognitively unimpaired (CU, *N* = 611, red), mild cognitive impairment (MCI, *N* = 531, green), and dementia (DEM, *N* = 210, blue), compared to the overall effect (black). ORs were derived from logistic regression models adjusted for relevant covariates. Cerebral microbleeds (CMBs) and white matter hyperintensity volume (WMHv) are shown to be significant predictors in varying degrees across cohorts. Lacunar infarcts were significant predictors in the CU cohort, only. Statistical significance determined by the Wald test is denoted as follows: *p < 0.05, **p < 0.01, ***p < 0.001.

We hypothesized that the presence of superficial CMBs would be associated with Aβ‐PET positivity across all cognitive stages. Examining each cohort separately, we identified a consistent and statistically significant relationship between superficial CMBs and Aβ‐PET positivity, as observed in the CU (OR = 1.38 [95% CI: 1.18–1.62], *p* < 0.001), MCI (OR = 1.37 [95% CI: 1.17–1.61], *p* < 0.001), and DEM cohorts (OR = 2.17 [95% CI: 1.46–3.29], *p* < 0.001; Table 3; Figure 1, panel 1).

**TABLE 3:** Adjusted cerebrovascular predictors of Aβ‐PET positivity by cohort.

Characteristic	OR^a^	95% CI^b^	*p*‐value
**CU cohort (*N* = 611)**			
CMBs, superficial (1+)	1.38	1.18, 1.62	<0.001
Infarction (1+)	1.31	1.09, 1.58	0.005
WMHv, normalized (log)	1.25	1.19, 1.31	<0.001
CMBs, deep (1+)	0.51	0.39, 0.66	<0.001
**MCI cohort (*N* = 531)**			
CMBs, superficial (1+)	1.37	1.17, 1.61	<0.001
CMBs, deep (1+)	1.32	1.02, 1.72	0.037
WMHv, normalized (log)	1.15	1.09, 1.21	<0.001
Infarction (1+)	0.89	0.73, 1.08	0.20
**Dementia cohort (*N* = 210)**			
CMBs, deep (1+)	2.99	1.36, 7.33	0.011
CMBs, superficial (1+)	2.17	1.46, 3.29	<0.001
WMHv, normalized (log)	1.54	1.33, 1.79	<0.001
Infarction (1+)	1.28	0.74, 2.28	0.40

*Note*: See Table S3 for the full model with covariates.

^a^ Adjusted odds ratios (OR) were adjusted for age, sex, *APOE* genotype, cognition (MoCA), and MTL volume.

^b^ CI, confidence interval; CMBs, Cerebral Microbleeds; WMHv, White Matter Hyperintensity Volume.

Conversely, when examining deep CMBs, we hypothesized that they would not significantly predict Aβ‐PET status for any cognitive stage. However, our analysis revealed differences in these relationships depending on cognitive stage: a negative association in the CU cohort (OR = 0.51 [95% CI: 0.39–0.66], *p* < 0.001) contrasted with a positive association in the MCI (OR = 1.32 [95% CI: 1.02–1.72], *p* = 0.037) and DEM cohorts (OR = 2.99 [95% CI: 1.36–7.33], *p* = 0.011; Table 3; Figure 1 **, panel 2**). These data indicate a potential complex relationship between deep CMBs and Aβ‐PET that appears to be dependent on cognitive stage.

### 3.4 Lacunar infarction

We next sought to characterize how lacunar infarction might correlate with Aβ‐PET positivity. Given the relatively low frequency of lacunar infarcts identified in our sample, we were unable to separately analyze infarct location or size‐dependent effects. Regardless, analyzing each cognitive stage separately revealed a significant association of one or more infarcts in the CU cohort (OR = 1.31 [95% CI: 1.09–1.58], *p* = 0.005), but not in the MCI (OR = 0.89 [95% CI: 0.73–1.08], *p* = 0.200) or DEM cohorts (OR = 1.28 [95% CI: 0.74–2.28], *p* = 0.400; Table 3 **;** Figure 1, panel 3). There was significant variability, highlighting a heterogeneous relationship between these lesions and Aβ‐PET.

### 3.5 White matter hyperintensities

We next investigated the predictive value of WMHv (normalized to whole brain volume) for Aβ‐PET positivity, postulating that the effects would be consistent across the disease course. We found that increased WMHv was a significant predictor of Aβ‐PET positivity across all disease stages: CU (OR = 1.25 [95% CI: 1.19–1.31], *p* < 0.001), MCI (OR = 1.15 [95% CI: 1.09–1.21], *p* < 0.001), and DEM (OR = 1.54 [95% CI: 1.33–1.79], *p* < 0.001; Table 3 **;** Figure 1, panel 4). These results remained significant after adjustment for other CVD measures, and additional covariates, indicating a consistent and strong relationship between WMHv and Aβ‐PET positivity across the cognitive spectrum, irrespective of MTL atrophy or whole brain volume.

### 3.6 Added benefit to assessing CVD biomarkers to predict Aβ‐PET positivity

To evaluate the influence of cumulative CVD burden on the likelihood of Aβ‐PET positivity, and to model how these biomarkers might be used in the context of a clinical workup, we next employed nested logistic regression models in our analysis of the overall cohort. Step 1 (Base) included participant demographics (age, sex), genetic screening (*APOE* ε4), and cognition (MoCA). Step 2 (Base + MTLv) included the same variables, with the addition of MTLv as a clinically relevant surrogate for AD‐related atrophy. Finally, Step 3 (Base + MTLv + CVD) incorporated our CVD biomarkers of interest (superficial and deep CMBs, lacunar infarcts, and WMHv). To compare the “added benefit” for each model step, we examined the change in AIC (Δ AIC) values between nested model steps, with negative values indicating improved parsimony in model prediction of Aβ‐PET positivity (i.e., improved goodness‐of‐fit without substantially increasing model complexity). For a list of these variables for each model step in tabular format, see Table S2.

In the overall cohort, our base model included age, sex, *APOE* ε4 genotype, and cognition (MoCA) as covariates (Step 1: pseudo‐R^2^ = 0.38; Table 4). As expected, we found that assessing MTL volume (MTLv), a commonly used measure of atrophy, enhanced model fit (Step 2: pseudo‐R^2^ = 0.39, Δ AIC = −11.43, d_3_[1] = 11.33, *p* = 0.001; Table 4). It is notable that further improvements in fit and parsimony were observed upon adding CVD biomarkers to the model, even when MTL atrophy had already been included (Step 3: pseudo‐R^2^ = 0.41, Δ AIC = −15.78, d_3_[4] = 5.30, *p* < 0.001). These findings indicate that for the overall cohort, inclusion of CVD biomarkers produced additional benefits to model fit without increasing complexity (i.e., the model became more parsimonious).

**TABLE 4 alz14207-tbl-0004:** Prediction of Aβ‐PET positivity with nested models in the overall cohort.

	Model 1: Base (Pseudo‐R^2^ = 0.38)			Model 2: Base + *MTLv^1^* (Pseudo‐R^2^ = 0.39)			Model 3: Base + MTLv^1^ + *CVD^2^* (Pseudo‐R^2^ = 0.41)		
Characteristic	OR^a^	95% CI^b^	*p*‐value	OR^a^	95% CI^b^	*p*‐value	OR^a^	95% CI^b^	*p*‐value
Age, years	1.08	1.07, 1.08	<0.001	1.07	1.06, 1.08	<0.001	1.05	1.04, 1.06	<0.001
Sex, male	0.67	0.62, 0.73	<0.001	0.66	0.60, 0.71	<0.001	0.66	0.60, 0.71	<0.001
*APOE* ε4, alleles	5.70	5.29, 6.15	<0.001	5.63	5.22, 6.08	<0.001	5.66	5.24, 6.11	<0.001
MoCA, score	0.86	0.85, 0.87	<0.001	0.89	0.88, 0.90	<0.001	0.89	0.88, 0.90	<0.001
MTLv, normalized (log)	–	–	–	0.10	0.07, 0.15	<0.001	0.12	0.08, 0.17	<0.001
WMHv, normalized (log)	–	–	–	–	–	–	1.25	1.20, 1.29	<0.001
CMBs, superficial (1+)	–	–	–	–	–	–	1.45	1.31, 1.61	<0.001
CMBs, deep (1+)	–	–	–	–	–	–	0.88	0.74, 1.04	0.120
Infarction (1+)	–	–	–	–	–	–	1.01	0.89, 1.15	0.900

*Note*: See Table S2 for description of nested model steps.

See Table S3 for nested models by cohort.

^a^ OR = odds ratio.

^b^ CI, confidence interval.

*N* = 1352.

Repeating this stepwise analysis across cognitive stages, model parsimony improved when CVD biomarkers, but not MTLv, were added to the workup of preclinical AD in the CU cohort (Δ AIC = −5.53, d_3_[4] = 3.22, *p* = 0.012). However, there was no consistent further benefit to prediction of Aβ‐PET positivity for the later disease stages when either MTLv or CVD biomarkers were added (Tables S3 and S4). Regardless, for the combined accessible model (i.e., ‘Step 3′: Base + MTLv + CVD), we observed moderate to large effect sizes for the prediction of Aβ‐PET positivity across all cohorts, with pseudo‐R^2^ values of 0.24, 0.37, and 0.45 in the CU, MCI, and DEM cohorts, respectively (Figure S1).

### 3.7 Exploratory analysis of diagnostic performance by cohort

We finally sought to explore the diagnostic performance of our combined accessible model in discriminating between Aβ‐positive and Aβ‐negative individuals with ROC analysis, using Aβ‐PET as the standard‐of‐truth. In the CU cohort, we identified a model AUC of 0.77 (95% CI: 0.75–0.78), consistent with moderate discriminatory ability between Aβ‐PET positives and negatives using the predicted probabilities estimated by the model. In the MCI cohort, we identified slightly improved discrimination between Aβ‐PET positives and negatives (AUC = 0.81 (95% CI: 0.80–0.83); in the DEM cohort, the model exhibited the highest discriminatory ability between Aβ‐PET positives and negatives (AUC = 0.90 [95% CI: 0.88–0.91]). Additional data exploring the diagnostic performance of the combined accessible model, including ROC curves and histograms with fitted probability values, are displayed in Figure S2 and Table S5.[Fig alz14207-fig-0001], [Table alz14207-tbl-0004]

## 4 DISCUSSION

In recent years there has been a growing emphasis on integrating multimodal biomarkers (genetic, fluid, and neuroimaging measures) to inform further workup of AD and to enhance the specificity of cognitive testing.^2^, ^33^ Our study extends this paradigm by incorporating accessible biomarkers of CVD into a model that already includes baseline demographics (age, sex), cognitive screening (MoCA), genetic information (*APOE* genotyping), along with clinically accessible neuroimaging measures of brain atrophy (MTL volume). These findings emphasize the underappreciated role of CVD biomarkers in AD diagnostics, and highlight their potential to provide a more holistic evaluation of AD and mixed pathologies.

Our findings underscore the need to consider each CVD biomarker distinctly in AD diagnostics and progression, a significant gap in the literature. For example, prior work indicates that the presence superficial “lobar” CMBs are highly specific to CAA.^15^ Our finding of superficial CMBs’ uniformly positive association with Aβ‐PET across cognitive stages aligns with prior studies and the Boston Neuroimaging Criteria 2.0, a widely accepted diagnostic strategy for CAA.^15^, ^34^ Deep CMBs (subcortical, periventricular, or brainstem) are less well characterized and may relate to mixed small vessel pathologies, including hypertensive vasculopathy.^35^, ^36^ However, we also found that deep CMBs displayed a biphasic relationship with Aβ‐PET positivity, marked by a negative association in the CU cohort, and a positive one in later stages of MCI and dementia due to AD. Although this requires further study, it is possible that mixed small vessel disease etiologies, affecting deeper brain regions, may explain the paradoxical positive association in MCI and dementia due to higher overall CVD burden contributing to clinically overt cognitive impairment.^5^, ^36^, ^37^, ^38^

In addition, WMH has been increasingly recognized as a marker of small vessel disease and is posited to contribute to cognitive decline in AD. Although mounting evidence links increased WMHv to Aβ, tau, and small vessel neuropathology, other studies suggest it may reflect AD‐related Wallerian degeneration in later disease stages.^24^, ^39^, ^40^, ^41^ Our findings of a consistent association between WMH and Aβ‐PET positivity across all cognitive stages suggest a stable and independent relationship with Aβ neuropathology. The reproducibility across cognitive stages, even after adjusting for other variables (including normalization of WMH to whole brain volume), further emphasizes WMH as a promising structural biomarker of Aβ pathology. This result echoes findings of longitudinal studies that link WMH to upstream Aβ accumulation and further progression of disease,^42^, ^43^ rather than a downstream side‐effect of Wallerian degeneration secondary to neurodegeneration associated with later disease stages. More work is needed to examine the performance of WMH alongside other CVD biomarkers in prediction of future disease progression.

Asymptomatic infarction is an understudied biomarker in AD and small vessel disease, due in part to challenges inherent in the variable size/distribution of pathology (cortical vs sub‐cortical/lacunar), unclear timing of injury (“silent” injury), as well as the underlying etiology (thrombotic, micro‐embolic, or watershed infarction).^16^, ^22^, ^44^, ^45^ Although we were not sufficiently powered to analyze these subtypes of stroke, or able to determine timing given the cross‐sectional design, taken together as a whole, we found that the presence of lacunar infarction was significantly associated with Aβ‐PET positivity in preclinical AD, but not in MCI or dementia groups. Whereas longitudinal analysis in a stroke cohort would be required to assess temporal relationships to Aβ positivity, our findings in preclinical AD are in line with basic science research linking stroke to Aβ deposition and impairment in perivascular Aβ clearance pathways as a potential driver of AD, rather than an incidental finding.^11^, ^46^, ^47^

Taken together, these results may facilitate the development of new CVD‐based assessments to aid in detection of Aβ neuropathology during preclinical and symptomatic stages, potentially increasing diagnostic confidence, especially important given the gravity of a dementia diagnosis.^3^, ^4^, ^5^ Because MRI is already used widely in the clinical workup of cognitive complaints,^18^ pending further validation and development of specific cutoffs for each measure, these CVD biomarkers could be translated to inform workup with blood biomarkers, Aβ‐PET, or cerebrospinal fluid analysis in a stage‐specific manner.^48^, ^49^, ^50^ Prior to translation to clinical use, more work is needed to validate this model in a data set that includes cases of vascular dementia alongside cases of “mixed” pathology and “pure” AD. This could enable development of AD‐specific cutoffs for each CVD biomarker; for example, milder CVD biomarker changes may be more consistent with AD, whereas more severe increases in CV burden could be more consistent with a primary diagnosis of vascular cognitive impairment. Based on our analysis, these optimal cutoffs may differ according to cognitive stage. Although additional studies are needed to address differentiation from vascular and other non‐AD dementias in clinical populations, our findings demonstrate the potential value of considering CVD biomarkers alongside established research criteria such as the National Institute on Aging–Alzheimer's Association Amyloid/Tau/Neurodegeneration Framework.^2^

Our cross‐sectional analysis has some important limitations, including potential ascertainment bias in the ADNI data set due to a high prevalence of Aβ positivity, which does not fully represent the spectrum of vascular or other non‐AD pathologies seen in clinical populations.^4^, ^44^ While excluding individuals with clear non‐AD pathology likely increases the specificity of our analysis, it may limit its generalizability to clinical populations with mixed pathologies. Survival bias may also be at play, given that cerebral and systemic vascular diseases are a leading cause of morbidity and mortality in older populations, potentially contributing to underestimation of the impacts of ischemic stroke in later disease stages.^51^, ^52^ Furthermore, the ADNI data set's relative lack of racial or ethnic diversity limits our findings, as neuropathological studies show a higher vascular co‐pathology burden in minoritized or traditionally underrepresented racial and ethnic groups.^3^ Taken together, these limitations may lead to an underestimation of the relationship between these CVD biomarkers and Aβ positivity.

Future research should focus on several key areas to build upon our findings. More work is needed to longitudinally assess these relationships, to better establish causality, and to develop and validate specific cutoffs that are tuned to clinical populations. Inclusion of additional CVD biomarkers, such as enlarged perivascular spaces and cortical/cerebellar microinfarction, may further improve diagnostic performance and potential specificity for AD.^53^, ^54^, ^55^, ^56^, ^57^, ^58^ Additional longitudinal studies are warranted to observe the progression of Aβ/tau pathology, neurodegeneration, and cognitive/functional decline over time in patients stratified by CVD biomarker status. These approaches could help define the bounds of AD, mixed vascular‐AD dementia, and primary vascular cognitive impairment. By gaining deeper insights into the trajectory of AD, it may also lead to improved prediction of outcomes with novel amyloid‐lowering immunotherapies, including risk of amyloid‐related imaging abnormalities (ARIA), a vascular consequence of treatment that underscores the need to better understand the relationship between CVD and AD. Implementation and standardization of CVD biomarkers could increase the efficacy and safety of these treatments via improved patient selection and monitoring protocols.^13^, ^59^, ^60^, ^61^

In summary, our study highlights the complex interplay between CVD and AD, pointing toward a more nuanced understanding of AD progression that incorporates accessible neuroimaging measures of CVD pathology. Although more work is needed prior to clinical use, this comprehensive analysis in a large observational cohort may help to refine diagnostic criteria for AD and informs future validation studies in more racially diverse and diagnostically complex cohorts. If successful, this integrative approach could lead to more personalized and effective treatment strategies, improving outcomes for individuals with AD.

## CONFLICT OF INTEREST STATEMENT

M. Howe: None to disclose. M. Caruso: None to disclose. M. Manoochehri: None to disclose. Z. Kunicki: None to disclose. S. Emrani: None to disclose. J. Rudolph: None to disclose. E. Huey: None to disclose. S. Salloway: Dr. Salloway has provided consultation to Biogen, Eisai, Avid, Lilly, Genentech, and Roche. H. Oh: None to disclose. Butler Hospital has received research grants from Biogen, Eisai, Avid, Roche, Genentech, Janssen, and Lilly. Author disclosures are available in the supporting information.

## CONSENT STATEMENT

Written informed consent was obtained from all participants or authorized representatives prior to the start of study procedures, with local institutional review boards and research ethics boards providing oversight for the study. The authors certify that the study was performed in accordance with the ethical standards as laid down in the 1964 Declaration of Helsinki and its later amendments. For more information, see adni.loni.usc.edu

## Supporting information

Supporting Information

Supporting Information
